# Axillary cat-scratch disease: A case report and literature review

**DOI:** 10.1097/MD.0000000000049722

**Published:** 2026-07-17

**Authors:** Cangsong Chen, Mingshuang Li

**Affiliations:** a Respiratory Department, Taizhou Hospital, Shanghai University of Traditional Chinese Medicine, Taizhou, Zhejiang, China; b Respiratory Department, Taizhou Hospital of Traditional Chinese Medicine, Taizhou, Zhejiang, China.

**Keywords:** axillary cat-scratch disease, case report, enlarged lymph nodes, fever, Warthin-Starry bodies

## Abstract

**Rationale:**

Cat-scratch disease (CSD) typically presents as self-limited regional lymphadenopathy following a cat scratch or exposure. Its clinical features can mimic malignancy or tuberculosis, especially in elderly patients, leading to potential misdiagnosis. This report underscores the importance of considering CSD in elderly individuals with unexplained fever and atypical lymphadenopathy.

**Patient concerns:**

A 67-year-old woman presented with a 3-month history of left axillary swelling, accompanied by pain and fever for 3 days.

**Diagnoses:**

Axillary CSD was confirmed based on clinical presentation, exposure history, and lymph node biopsy findings.

**Interventions:**

The patient received compound sulfamethoxazole tablets as anti-infective therapy.

**Outcomes:**

Two weeks post-treatment, the patient was afebrile, and the surgical incision had healed.

**Lessons:**

This case highlights the diagnostic challenges of CSD in the elderly. Early lymph node biopsy and careful elicitation of exposure history are essential to avoid misdiagnosis, particularly with malignancy. Timely intervention can prevent unnecessary procedures and improve prognosis. Evaluation of confirmed cases should also include an assessment for potential extra-nodal spread to sites such as the liver, colon, and retina.

## 1. Introduction

Cat-scratch disease (CSD), or benign lymphoreticulosis, is a zoonotic infection primarily caused by *Bartonella henselae*. It typically presents as self-limited regional lymphadenopathy in the drainage area of an inoculating cat scratch or bite.^[1]^ Bartonella is a zoonotic pathogen that can be transmitted from mammals to humans via a variety of vector insects such as cat fleas, body lice, ticks, and chiggers. The escalating global prevalence of pet ownership has been paralleled by a rise in the incidence of CSD. Nevertheless, epidemiological data in China remain limited, and the condition continues to pose persistent diagnostic challenges in clinical practice.^[2]^ Recent studies have indicated that the occurrence of the disease is closely related to climate. Climatic factors may indirectly affect the occurrence and spread of diseases by influencing the ecological distribution of hosts, vectors, and pathogens.^[3]^ CSD, while most common in adolescents, typically presents with a primary inoculation papule, followed by regional lymphadenopathy, which may be accompanied by systemic symptoms such as fever, malaise, and abdominal pain. Importantly, severe disseminated disease can occur, involving the visual, nervous, or cardiovascular systems. Due to its nonspecific clinical presentation, It poses a significant risk of misdiagnosis and inappropriate treatment, which can, in severe cases, lead to life-threatening complications.^[4]^ This case report describes an elderly female patient with CSD who demonstrated significant clinical improvement following appropriate antimicrobial therapy.

## 2. Case introduction

**Figure 1.**
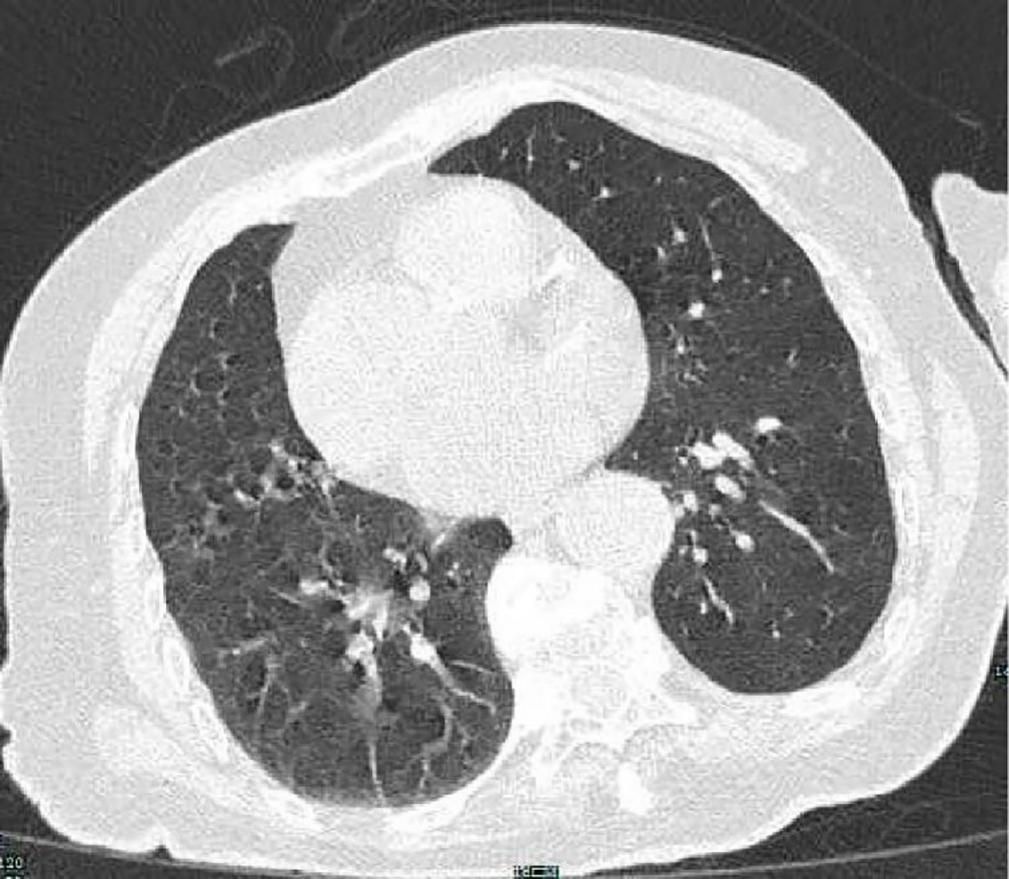
The chest CT scan revealed no tumors or tuberculosis, showing only bronchial lesions.

**Figure 2.**
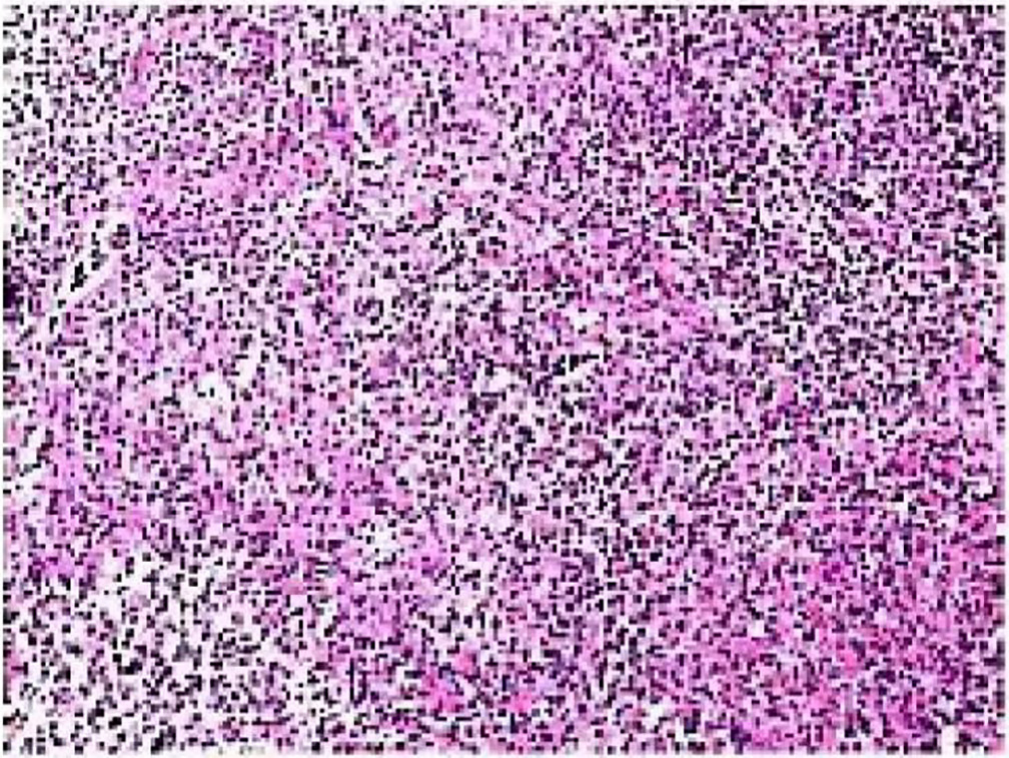
HE staining of lymph node tissue. The results suggest granulomatous inflammation of the lymph nodes with neutrophilic infiltration (HE stain: original magnification × 100).

A 67-year-old female patient presented with complaints of left axillary pain and fever. She had previously been in good health and denied any significant medical history. Three months prior to presentation, she first noticed a painless, approximately 1cm × 1 cm mass in her left axilla. The lesion was not associated with tenderness, skin changes, or other discomfort, and she had not sought medical evaluation at that time. The clinical picture changed 3 days before admission when she developed left axillary pain accompanied by chills and fever. The patient’s systemic review was negative for upper respiratory symptoms (rhinorrhea, nasal congestion, sore throat, cough), as well as cardiopulmonary (chest tightness, pain) and gastrointestinal complaints (abdominal pain). Prior to admission, laboratory tests at a local hospital revealed a normal WBC count (8.26 × 10^9^/L) with elevated neutrophils (75.2%) and an elevated CRP (C-reactive protein) level (20.77 mg/L). An axillary ultrasound had identified several hypoechoic masses bilaterally, with multiple enlarged lymph nodes on the left side. After 2 days of oral administration of cefdinir (100mg tid) for anti-infective treatment failed to resolve her symptoms, with persistent fever peaking at 38.6°C, she was admitted on July 2, 2024 for further evaluation. Upon admission, her vital signs documented a fever of 38.3°C, and on physical examination, appeared thin. Palpation of the left axilla confirmed a 2.0 cm × 1.0 cm mass that was mobile, tender, and of moderately firm consistency with intact overlying skin. Repeat laboratory investigations at admission showed WBC and Neutrophils within normal range, with a markedly elevated erythrocyte sedimentation rate (81 mm/h) and serum ferritin level (211.1 μg/L). Serological tests for syphilis and human immunodeficiency virus were negative, and tumor markers were within normal limits. A chest CT scan was performed to exclude pulmonary tuberculosis or malignancy, which ruled out significant pulmonary nodules, masses, or cavities, showing only nonspecific bronchial abnormalities (Fig. 1). Ultrasound examination of the liver, gallbladder, spleen, and pancreas showed no abnormalities. After receiving anti-infective treatment with cefotaxime (2g, intravenous drip, twice a day) for 3 days, her body temperature remained elevated between 37.3°C and 38.4°C, and the left axillary pain persisted. After repeated inquiries about the medical history, the patient reported a history of cat-scratch injury Additionally, the patient tested positive for Pasteurella multocida serological immunofluorescence antibody (IFA) IgM (>1:40). To clearly differentiate from breast cancer, a left axillary lymph node dissection biopsy was performed. To establish a definitive diagnosis, left axillary lymph node clearance biopsy was performed. The surgical exploration revealed multiple fused axillary lymph nodes with a friable texture and borders that were adherent to the surrounding tissues. An approximately 2.0 cm × 1.5 cm node was selected and carefully excised. Pathological examination confirmed lymph node granulomatous inflammation with neutrophilic infiltration (Fig. 2). The therapeutic regimen was subsequently adjusted to oral compound sulfamethoxazole tablets (Sulfamethoxazole 800 mg/Trimethoprim 160 mg, twice daily) for 5 days. The patient was discharged in improved condition. At the 2-week follow-up, she was asymptomatic with a well-healed incision and no evidence of inflammation. A brief timeline of the hospitalization was provided in Table 1.

**Table 1:** Brief timeline of events during patient’s hospitalization

Date	Symptom	Treatment
July 2, 2024–July 4, 2024	Fever (T max 38.4°C), axillary pain did not improve.	Cefotaxime (2 g, intravenous drip, twice a day)
July 5, 2024–July 6, 2024	Fever (T max 38.0°C), axillary pain did not improve.	Cefotaxime (2 g, intravenous drip, twice a day) + compound sulfamethoxazole tablets (Sulfamethoxazole 800 mg: Trimethoprim 160 mg, po, twice a day)
July 7, 2024	Fever (T max 37.5°C), axillary pain was partially relieved.	
July 8, 2024	No fever (T max 36.9°C), axillary pain was partially relieved.	
July 9, 2024	No fever (T max 36.4°C), axillary pain has significantly improved.	

## 3. Discussion

CSD, transmitted to humans through scratches and bites from cats, is also known as cat-scratch fever or subacute regional lymphadenitis. This disease is typically self-limiting and usually resolves spontaneously in immunocompetent hosts following infection. Most patients present with unilateral involvement affecting a single lymph node. A minority may develop multiple lymph node involvement. The infection can spread hematogenously to the visual system, causing optic nerve retinopathy and inflammatory optic disc edema. Epidemiology from the United States, Europe, Australia, and Japan has shown that CSD has a worldwide distribution.^[5]^ A retrospective study in the United States showed a seasonal distribution of the disease, with peak incidence in January and late summer to fall.^[6]^ The diagnosis of CSD should meet 3 of the following 4 criteria^[7]^: a history of cat or dog scratching and biting, contact, or local tissue damage; a positive specific antibody test or specific antigen skin test; lymph node pathology showing characteristic granulomatous lesions; and the exclusion of other diseases, such as tuberculosis, lymphoma, and lymph node inflammation. This case involves an elderly woman who initially presented with weight loss and an axillary lump, without chills or fever. Subsequently, she developed fever, accompanied by axillary pain and axillary lymphadenopathy. Due to the lack of specificity in clinical manifestations, CSD is easily confused with several other diseases. Despite the presence of fever and granulomatous inflammation, pulmonary tuberculosis was ruled out by the absence of radiographic findings, systemic symptoms, and exposure history. Occult breast cancer is a distinct clinical entity characterized by the presence of metastatic carcinoma, most commonly in axillary lymph nodes, without a detectable primary breast lesion.^[8]^ The diagnosis is ultimately established through pathological confirmation. Hodgkin lymphoma classically manifests with painless peripheral lymphadenopathy and intermittent fever. The diagnosis is histologically defined by the presence of characteristic Reed-Sternberg (R-S) cells on lymph node biopsy.^[9]^ Sporotrichosis often presents as cutaneous lymphangitis with ulcerative nodules. A confirmed diagnosis relies on culture, but histopathological examination aids in identification.^[10]^ After repeated questioning, the patient reported that she had been scratched by a cat 3 months ago. The absence of Worsham-Staley staining or PCR results is the limitation of this study. However, the patient’s exposure history, clinical presentation, and response to treatment all support this diagnosis. CSD is a self-limiting disease, and does not require drug treatment. In severe cases such as high fever, encephalitis, and immunodeficiency, early treatment with antibiotics can prevent the infection from aggravating. Clinical treatment is generally combined with antimicrobial drugs, such as rifampicin, erythromycin, doxycycline, cotrimoxazole, and so on. The course of treatment is more than 2 weeks, and puncture of the affected lymph nodes for pus or resection can be considered if necessary. This case underscores the diagnostic challenges of CSD, particularly when initial empirical therapy does not cover Bartonella henselae. The initial empirical therapy with cefotaxime, which has poor activity against *Bartonella henselae*, proved ineffective. A definitive diagnosis was subsequently established by lymph node biopsy, which prompted a revision of the treatment regimen to include compound sulfamethoxazole. The patient’s condition improved promptly thereafter, highlighting the essential role of timely histopathological evaluation in diagnosing atypical lymphadenopathy.

In summary, the diagnosis of CSD should be based on a combination of medical history, clinical manifestations, and lymph node biopsy pathology. In patients with suspected CSD, lymph node biopsy should be considered to achieve an early confirmation of the diagnosis, thereby facilitating timely treatment and potentially shortening the disease course. Furthermore, reinforcing public education on the health risks of pet scratches is crucial for primary prevention. Concurrently, clinicians should maintain a high index of suspicion for CSD, particularly in cases of unexplained lymphadenopathy. A multidisciplinary approach and the comprehensive application of diagnostic tools are essential to ensure timely identification and appropriate management.

## 4. Conclusion

The case report described an elderly female patient with CSD whose atypical clinical presentation leads to delayed diagnosis. Early biopsy and detailed patient history are critical in elderly patients with axillary lymphadenopathy to avoid misdiagnosis of CSD as malignancy. According to the basic immune status and affected tissues, timely and reasonable use of antimicrobial drugs can lead to a good prognosis.

## Author contributions

**Conceptualization:** Cangsong Chen, Mingshuang Li.

**Investigation:** Cangsong Chen.

**Writing – original draft:** Cangsong Chen.

**Writing – review & editing:** Mingshuang Li.
